# 'What do doctors think they need to know about nutrition?’—a qualitative study of doctors with formal nutrition training

**DOI:** 10.1186/s40795-022-00577-w

**Published:** 2022-08-22

**Authors:** G Caldow, C Palermo, AN Wilson

**Affiliations:** 1 Melbourne Medical School, Faculty of Medicine, Dentistry and Health Sciences, University of Melbourne, Melbourne, Australia; 2 Monash Centre for Scholarship in Health Education, Monash University, Melbourne, Australia; 3 Maternal, Child and Adolescent Health Program, International Development, Burnet Institute, Melbourne, Australia

**Keywords:** Qualitative study, Nutrition education, Medical education, Social determinants

## Abstract

**Background:**

Doctors are on the frontline of patient care and in an ideal position to provide nutritional advice, yet can feel ill-equipped to do so. The aim of this study was to explore the nutrition knowledge, skills and practice required for nutrition-competent medical graduates, and their role in providing nutrition advice and care, from the perspective of doctors with formal nutrition training.

**Methodology:**

We conducted an exploratory qualitative research study. A purposive sample of 12 medical doctors and students with formal nutrition training across Australia participated in in-depth semi structured interviews. Data were analysed thematically.

**Results:**

There were four main themes identified: 1. Identifying the role of doctors in nutrition care; 2. Understanding the interrelatedness of the social determinants of health and nutrition status is key; 3. Optimising nutrition care through multidisciplinary collaboration; and 4. Providing evidence-based nutrition care.

**Conclusion:**

This exploratory study suggests that doctors consider that nutrition competent medical graduates require skills in referring to dietitians, an understanding and application of the social determinants of health, and practise applying multidisciplinary and evidence-based nutrition care.

## Introduction

Nutrition is essential for human health and wellbeing and associated with social and economic development [1]. In response to the need for more global action on nutrition and all forms of malnutrition, the United Nations declared 2016–2025 as the decade of action on nutrition [2].

Doctors are ideally placed to provide nutrition care [3]. However, medical practitioners can feel ill-equipped to provide this care, due to a lack of nutrition knowledge and skills in providing nutrition advice [4]. Nutrition education in medical curricula is limited [4]. There is a paucity of nutrition accreditation standards for medical curricula [5], and no consensus on the knowledge and skillset required by doctors for competent nutrition care [4]. Directives for nutrition education vary and the role of doctors in nutrition care remains undefined. Previous studies have recommended doctors require specific nutrition knowledge to address non-communicable diseases, whilst others emphasise referral skills and teamwork [5]. Many published studies exploring the role of nutrition education in medicine have been from the perspective of medical students, doctors, dietitians and medicine faculty members [4–8]. This ambiguity in nutrition medical education is reflected in practice, doctors rarely deliver dietary interventions and referral to dietitians is low [9]. Without a strong foundation in nutrition education and a clearly defined role, doctors may not have the skillset nor understanding to prioritise nutrition care. The perspectives of what curricula is required from doctors with experience in medicine and nutrition, who understand the constraints of both worlds, has not been previously explored.

This study aimed to explore the nutrition knowledge, skills and practice required of nutrition-competent medical graduates and their role in providing nutrition advice from the perspectives of medical students and doctors with formal nutrition training.

## Methodology

### Methodological approach

A qualitative study with an interpretive research approach. Given the small number of doctors with both medical and nutrition qualifications in Australia, interpretivism was useful in exploring perspectives of this small sample. Their unique experiences, perspectives and behaviours may support the contextualization of the findings to medical education more broadly.

### Setting, participants and procedures

Participants were purposefully sampled as Australian doctors or medical students with nutrition qualifications. University nutrition and dietetics qualifications (for example, Bachelor or Masters degrees in Nutrition and/or Dietetics) as well as post-graduate fellowships in lifestyle medicine (for example, nutrition education provided by https://www.lifestylemedicine.org.au/fellowship/) were deemed eligible nutrition qualifications. Participants were recruited through two authors' professional networks to identify medical students/doctors with formal nutrition training, and also through nutrition in medicine special interest groups. Snowball sampling was also used to recruit further participants [10]. In total, 17 medical students/doctors were initially identified, and invited to participate in the study by email invitation. There were 15 responses to these invitations, however three participants were unable to participate due to schedule clashes.

### Data collection

Participants who consented to participate in the study were provided with a demographics survey, which included questions on their nutrition qualifications and medical specialty. Participants were then invited to undertake in-depth semi structured interviews. Interview questions were designed based on a preliminary analysis of the literature on what medical graduates need to know about nutrition [5–14]. The semi-structured interview guide facilitated discussions (Table 1) and enabled exploration of participants’ training in nutrition, and perspectives and experiences of the role of medical doctors in nutrition care. In addition, participants were asked to provide their suggestions for improving the nutrition content of medical curricula.

**Table 1:** Interview guide for semi-structured interviews

Line of inquiry	Questions
Introduction	1.Can you tell me a little about yourself and your career progression? 2.Tell me about what study/training you have undertaken in nutrition? 3.What thoughts, feelings or perspectives come to mind when you think about your nutrition training?
Unique perspectives from working in two professions	4.Tell me about how your further study/training in nutrition informs your current work? 5.Tell me about the nutrition knowledge and skills that you regularly draw on in clinical practice? What supports (or hinders) you being able to use these skills in clinical practice? 6.What are some of the challenges in providing nutrition care in your current role? What would nutrition focused medicine look like in an ideal world? 7.In an ideal scenario how would a doctor work effectively with a dietitian to optimise nutrition outcomes for a client/patient?
Directions for medical curriculum	8.Tell me about what nutrition education you received in medical school? What were the strengths? Limitations? 9.If applicable, what limitations do you recognise in the current medical curriculum? 10.What do you think that doctors need to know about nutrition? 11.What nutrition knowledge and concepts do you think are essential in medical education? 12.What skills and abilities do doctors need to have upon entry to internship? OR What nutrition competencies are essential for medical graduates? How should competency in nutrition be assessed in medical school? 13.How should nutrition education be taught in medical school and further training? 14.Do you have any perspectives on who is best placed to teach nutrition? 15.What are the current gaps in nutrition skills in the medical workforce, and how may these gaps be rectified?

Interviews were initially conducted face-to-face at a mutually convenient venue (i.e. café or workplace) or online via the online platform Zoom, depending on participant preferences. However, as a result of the COVID-19 pandemic and associated restrictions, interviews were moved to solely over the phone/online. All interviews were conducted between February and June 2020. The interviewer was a doctor in training with an interest in nutrition who was not known to participants. Interviews were conducted until recruitment of doctors and medical students with formal nutrition training identified from authors’ professional networks were exhausted, and there was sufficient information power to answer the research question in line with our interpretive approach [10]. Member checking was conducted during interviews whereby the interviewer would restate or summarise information provided by the participant back to the participant to determine the accuracy of the data recorded. All interviews were audio recorded and transcribed verbatim.

### Data analysis

NVivo version 12 Qualitative Data Analysis software (QSR, International 2018) was used to support the data analysis. Transcripts were initially read several times over before being coded. Initially two members of the research team independently manually coded two transcripts each. Data was inductively coded by labelling units of text. These two authors came together to compare and discuss the codes. A codebook was developed with the agreed codes which were then entered into NVivo, and applied to the remaining transcripts, with an iterative approach used to allow identification of new codes. All transcripts were then coded by the first author (GC). Once all transcripts were coded, the codes were then grouped into categories. The categories were considered in light of the research question to identify prominent themes.

### Data trustworthiness

Categories were compared across participant groups to identify different patterns in the data from those with dietetics qualifications compared to other qualifications. Coding patterns and emergent themes were discussed and refined in ongoing meetings between team members. Field notes were referred to during the analysis to facilitate theme development. Prolonged engagement with the data allowed research immersion and ensured credibility [15]. Reflexivity was practiced whereby researchers challenged their interpretations based on their own experiences. Team member debriefing exercises throughout the research process facilitated confirmation of prominent themes.

### Ethics

Ethics approval was provided by the Melbourne University Human Research Ethics Committee (Ethics ID: 1.955.671.1).

## Results

Twelve participants agreed to participate and took part in the study (Table 2). Of these participants, nine were doctors and three were medical students. In addition, eleven (92%)  of these participants identified as female and one as male. The majority (42%) of participants were aged between 25-34 years. Those who had completed their medical specialty training had between five  to 10 years of medical experience (58%). Most were General Practitioners (58%) and had completed a Bachelor of Nutrition and Dietetics (58%) prior to commencing their medical studies.

**Table 2:** Interview participant demographics

Demographic	Category	Number of participants (total) n(%) *n* = 12
**Age (years)**	18–24	1 (8%)
	25–34	5 (42%)
	35–44	4 (33%)
	45–54	1 (8%)
	55–64	1 (8%)
	> 65	0
**Gender**	Female	11 (92%)
	Male	1 (8%)
**Nutrition training**	Bachelor of Dietetics and Nutrition	7 (58%)
	Bachelor of Nutrition Sciences/Food Sciences	1 (8%)
	Bachelor of Pre-medicine, Science and Health (Nutrition)	1 (8%)
	Fellow Australasian Society of Lifestyle Medicine	1 (8%)
	E-Cornell Certificate of Plant Based Nutrition	2 (17%)
**Years of medical experience (years)**^1^	< 5	1 (85)
	5–10	7 (58%)
	11–15	1 (8%)
	16–20	0
	21–30	0
	> 30	1 (8%)
**Medical Specialty**^2^	General Practice	8 (67%)
	Emergency Medicine	1 (8%)
	Obstetrics and Gynaecology	1 (8%)
	Student	2 (17%)

^1^Students were not included in this variable

^2^One General Practitioner referred to themselves as an Integrative and Lifestyle Medicine Specialist

The qualitative analysis identified four key themes which are outlined below and further illustrated through quotes provided.

### Theme one: Identifying the role of doctors in nutrition care

Participants expressed mixed views regarding the role of doctors in nutrition care. Those with a dietetics qualification were more likely to perceive the role of doctors as facilitating referral to dietitians. *“I guess I see my role is more just like this ability to facilitate a referral. And knowing [how] to you know, hopefully make that referral as early as possible to help out the dietitian to be able to provide the best care.”* (P7, Dietetics background).

Recognising when and knowing how to refer to dietitians was prioritised over specific nutrition knowledge. Those who prioritised referral recognition believed there were strict roles for different health professionals in providing nutrition care and perceived that dietitians were the sole providers of nutrition care, not wanting to ‘*step on the toes’* of dietitians (P7, Dietetics background).

Participants highly prioritised nutrition care from practising dietitians rather than providing nutrition care directly themselves, as one participant illustrated when describing how they would describe this to a patient, *“Now I'm going to refer you to the dietitian who is going to spend more time and give you current, up to date information. And I want you to follow that dietitian’s advice because they’re the experts or specialists in this”* (P10, Dietetics background).

Despite having formal training in nutrition, some participants expressed a lack of confidence in providing nutrition care, citing lack of practice, time and dated nutrition knowledge. For these participants, confidence and having a role in providing nutrition care seemed to go hand in hand, the less confident participants felt, the more likely they were to refer to dietitians.

*“I refer to dietitians a lot. I don’t think I can replicate what they do; partly because I’m not practiced, and partly because I don’t have the time.” *(P11, Dietetics background)

Conversely, other participants believed that doctors should provide prescriptive nutrition advice to patients as part of holistic care. These participants noted that doctors’ advice is highly regarded by patients and as such they have a responsibility to provide nutrition advice.

*“It's that reinforcement that you're all giving that same message. Which I think is really helpful. Because when people get mixed messages, they get really confused, and they don't know who to believe.” *(P5, Dietetics background).

### Theme two: Understanding the interrelatedness of the social determinants of health and nutrition status is key

Participants with a dietetics background noted that doctors require a better understanding of the social determinants of health when providing nutrition care. These participants noted a lack of content in medical school curricula regarding the impact and interrelatedness of the social determinants on dietary choices, nutrition status and health.

*“I think [in the dietetics degree] we had like a whole semester subject on sort of the social determinants of health and nutrition. Like, what influences that and we did a lot of activities, you know, where we looked at what influences our own diet choices and what might influence other people and I think that's probably really poorly understood amongst medical people” *(P3, Dietetics background).

Many participants observed a lack of empathy from medical doctors for a patient’s social, emotional or health status, particularly in the context of obesity, with physicians minimizing symptoms such as muscle pain due to bias that this was caused by poor patient dietary choices. *“It’s just this very complex mix of things; reasons why they may be overweight and it’s not… I don’t know, they’ve eaten too much and been lazy, whatever the general stigma is. Sometimes you hear people say things and it’s quite obvious that they’re lacking of a bit of compassion.”* (P12, Dietetics background).

Participants also reported that a lack of understanding of the social determinants of health may leave patients feeling stigmatized, and result in a lack of trust in the medical profession.

*“I actually think it's no wonder that like, a lot of patients sort of have recently been coming out publicly like ‘why does the doctor weigh me every time when I go for a cold? Why are they telling me to lose weight?’ And I actually don't think it's an overreaction. I see it all the time.” *(P3, Dietetics background).

### Theme three: Optimising nutrition care through multidisciplinary collaboration 

Participants reported a lack of collaboration between doctors and allied health professionals. Many urged for stronger professional relationships between doctors and dietitians to ensure continuity of care. *‘More importance needs to be put on the support factor of doctors and GPs, the cohesiveness of the team, the communication between the team including allied health.’* (P1, associated with a nutrition special interest group).

Those with a dietetics background noted that collaboration between doctors and dietitians was limited and that both parties had pre-conceived ideas and attitudes towards each other’s disciplines, citing a lack of understanding and respect of the skillset of each occupation.

*“It's really interesting when people or how people change their tune, communicating with me as a medical student, say, like nurses or allied health when I like tell them my background.” *(P7, Dietetics background)

### Theme Four: Providing evidence-based nutrition care

Participants believed that nutrition education and advice when given by doctors must be evidence based. *“I think because doctors respond to evidence base there’s a few… I don’t know, presenting and finding a few landmark studies about what it can do for your risk of cancer long term, what it can do for your risk [of] heart disease long term, longevity, that kind of thing.”* (P12 Dietetics background).

There was concern about nutrition practice occurring outside of the evidence base, with stories of their medical colleagues promoting ‘fad’ or non-evidence based diets. When prompted for a solution to this, many recommended that graduates should have the skills to critically appraise nutrition literature, just as they would other aspects of medicine, and always provide evidence-based care.

*“I've, you know, seen a doctor personally who's a bit like, ‘Ah, you know, keto is really good. You should try keto.’ But actually, when you look at it it’s like, A. It’s not sustainable. And when you're making dietary change, you really want it to be sustainable, and all that sort of stuff. And B. probably not great because there’s a lot of saturated fats and that sort of stuff. Like you might get the weight loss, but at what cost?” *(P3, Dietetics background)

## Discussion 

This is the first study to explore what doctors need to know about nutrition and their role in nutrition care from the perspectives of medical doctors with additional formal training and/or advanced education in nutrition. It has highlighted different perspectives regarding the role of doctors in nutrition care, the importance of referrals to dietitians, the need for skills in addressing the social determinants of health and that nutrition care must be a collaborative effort based on the evidence.

Many participants perceived that doctors should play an active role in facilitating referrals to dietitians, and that providing nutrition advice fell outside of doctor’s responsibilities. Conversely, previous literature has suggested that doctors are well positioned to provide nutrition advice to patients[3], and access to dietitians can be limited due to financial and geographical issues[16]; with a higher density of dietitians placed in urban centres. Existing directives for nutrition education in medicine, such as a position from the American Heart Association[12], and the Nutrition Education in Medical Schools (NEM) paper[13], have focused on specific knowledge of topics such as micronutrients, macronutrients, metabolism and nutritional intervention for lifestyle disease[4]. In contrast, our study emphasised the importance of referral skills and reinforcing nutrition advice provided by dietitians. This may reflect a lack of confidence in providing nutrition care or reflection on dietitians’ scope of practice. In addition, our participants’ responses may be reflective of their exposure to dietetics, whereby these participants may better understand the specialist knowledge of dietitians, and their own personal limitations and time constraints as a doctor in providing up to date and clinically relevant advice. Medical curricula are often considered to be ‘overcrowded’[4]. Our findings suggest that more complex nutrition may not need to be covered, but an understanding of allied health specialty areas and skillsets, as well as an emphasis and promotion of referral skills to dietitians in clinical practice is important, where time or expertise is not available to the medical practitioner.

Previous qualitative research has suggested that some physicians may not speak respectfully to allied health professionals, ignore their recommendations, and fail to genuinely participate in multidisciplinary discussions [18]. Open and frequent multidisciplinary discussions between medical teams and allied health is needed to build mutual respect and encourage collaboration between multiple disciplines. Our study finding that nutrition care is optimised through multidisciplinary collaboration is consistent with the existing literature [19–21] A study which aimed to explore the benefits of multidisciplinary collaboration, involved registered dietetic students’ coaching medical students on giving nutrition advice to patients. This was found to be an effective learning activity in developing skills and inter-professional connections [22]. In planning medical curricula, learning activities that promote multidisciplinary collaboration may be efficacious in facilitating medical graduates who appreciate the role of allied health professionals and know when and how to refer patients appropriately.

Participants noted the importance of the social determinants of health in providing nutrition care. Many reported that doctors may not always consider the complexity of social factors which contribute to a patient’s nutritional status, and this can leave patients feeling stigmatised. For example, in our study participants discussed instances where doctors discussed weight management with a patient regardless of their presentation, such as, offering weight loss advice for a patient with a common cold. Despite the widespread inclusion of the social determinants of health in medical education, these messages do not necessarily translate into practice. This was explored in a perspective piece that found that current implementation of the social determinants of health in curricula does not necessarily lead to outcomes that reduce inequity [23]. For example, learning objectives around the social determinants are often to know about them, rather than giving student’s skills to address the underlying causes that create inequity, such as racism, oppression, sexism and social and economic power imbalances [23]. In order to practice nutrition care that leads to improved health outcomes, medical graduates need to be equipped with the skills to address and challenge barriers to optimising the social determinants of health together with different health disciplines including dietitians.

The need for doctors to promote and practice evidence based nutrition care was noted by many participants. Participants had observed doctors promoting non-evidence based or ‘fad’ diets and noted that medical graduates require an understanding of the evidence to provide evidence-based dietary advice. However, there was wide variability in what participants considered evidence based. For example, those who were part of a special interest group suggested evidence that supported their interests including plant-based diets and integrative medicine such as incorporating naturopathic practices with nutrition evidence, use of supplements, and the health at every size (HAES) approach to nutrition. Nutrition education needs to be evidence-based with key landmark studies, such as, the PREDIMED Mediterranean Diet study [24], to ensure the understanding and provision of evidence based care. Our study findings recommend that medical graduates receive skills to critically appraise literature in nutrition, as well as medicine. The NNEdPro Global Centre for Nutrition and Health, Foundation Certificate and Summer School in Applied Human Nutrition [25], is an existing nutrition course that includes critical appraisal training and education. These skills allow medical graduates to continue to evaluate dietary advice across their career.

Our study findings are relevant to existing nutrition directives, such as the UK Association for Nutrition Undergraduate curriculum [26]. For example, the social determinants of health in the context of nutrition may be incorporated into teaching and discussed through case studies and problem-based learning exercises. In addition, there should be opportunities for medical students, during skills based tutorials, to practise their referral skills in making a referral to a dietitian, as well as referrals to other allied health professionals. Multidisciplinary collaboration and networks could be established through opportunities for medical students to undertake projects and assessments with students from other health disciplines, which has been identified as a successful approach in other settings [22].

The limitations of this study include the small sample with limited diversity across nutrition qualifications. Having a greater balance of participants with alternate nutrition training may have deepened the findings. This study was unable to explore differences between gender as only one participant identified as male. We also did not look into geographical differences that may exist due to rurality. A strength of this study was that participants had a unique lived experience as nutrition and medical practitioners. Participant’s enriched experience demonstrated understanding of both medical and dietetics professions.

Further research is essential to identify and create standardised nutrition education in medical curricula that is relevant, prioritised and can be measured objectively to ensure improved patient outcomes and holistic care. Incorporation of referral skills, literature appraisal and team based care are concepts that can be readily incorporated into the curricula through problem based learning and practical skills sessions. Furthermore, increased focus on how to address the social determinants of health should be reinforced with teaching of clinical presentations. Current medical practitioners would benefit from this approach to nutrition education and ideally provide more holistic health care as a result.

## Conclusion

This study has illustrated that competent nutrition care from doctors goes beyond nutrition knowledge. Whilst doctors need to have a basic understanding of nutrition to work collaboratively with dietitians and nutritionists, there equally needs to be an understanding of the social determinants of health that influence dietary patterns, nutrition and health more broadly. Nutrition care provided by doctors should be multidisciplinary and evidence based. Learning opportunities that promote team based care with allied health professionals are essential. Enhancing medical education now and into the future requires dedicated investment and resources to build the next generation of nutrition competent doctors.

## Data Availability

Due to the involvement of specific participants in this study – medical doctors with former education and training in nutrition and/or dietetics – there is a high risk of identification with the public provision of qualitative data. As such, datasets are not publicly available due to confidentiality concerns, however additional information can be made available from the Scientific Integrity Officer at Burnet Institute, (admin@burnet.edu.au), on reasonable request.
